# Catalogue of the Medical Department of the Cincinnati College

**Published:** 1838

**Authors:** 


### EXTRA-LIMITES.

---

Art. I.—Catalogue of the Medical Department of the Cincinnati College, for 1837–8.

### FACULTY.

The Rev. WILLIAM HOLMES McGUFFEY,
*President of the Institution.*

JOSEPH N. McDOWELL, M. D.
*Professor of Special and Surgical Anatomy.*

SAMUEL D. GROSS, M. D.
*Professor of General and Pathological Anatomy and Physiology.*

WILLARD PARKER, M. D.
*Professor of Surgery.*

LANDON C. RIVES, M. D.
*Professor of Obstetrics and the Diseases peculiar to Women and Children.*

JAMES B. ROGERS, M. D.
*Professor of Chemistry, and Medical Jurisprudence, and Dean of the Faculty.*

JOHN P. HARRISON, M. D.
*Professor of Materia Medica and Pharmacy.*

DANIEL DRAKE, M. D.
*Professor of the Theory and Practice of Medicine.*

CARY A. TRIMBLE, M. D.
*Anatomical Demonstrator.*

### CATALOGUE OF STUDENTS.

### THIRD SESSION.

| NAMES. | RESIDENCE. | PRIVATE PRECEPTORS. |
|---|---|---|
| William E. Akin, | *Kentucky,* | Drs. Fleece & Weisiger. |
| Nathaniel S. Armstrong, | *Ohio,* | Prfs. Harrison & McDowell. |
| John S. Alexander, | *Kentucky,* | Prf. Gross. |
| **B** | | |
| Julius O. Beardslee, | *Ohio,* | |
| Israel Brown, | *Ohio,* | Prf. Gross. |
| William Bobbs, | *Ohio,* | Dr. Blymiger. |
| Mahlon Black, | *Ohio,* | Dr. Jewett. |
| Littleton E. Bennett, | *Kentucky,* | Dr. Bennett. |
| Thomas J. Bicknell, | *Virginia,* | Dr. Tanner. |
| John Black, | *Alabama,* | Dr. McDonald. |
| James V. Boughner, | *Pennsylvania,* | Dr. Stephenson. |
| John W. Brookbank, | *Indiana,* | Dr. Casterline. |
| James A. Brown, | *Missouri,* | Prfs. Harrison & McDowell. |
| **C** | | |
| William P. Cushman, | *Ohio,* | Dr. Colby. |
| Lewis H. Carey, | *Ohio,* | Dr. R. E. Lord. |
| Ziba Casterline, | *Indiana,* | Practitioner. |
| Preston Capshaw, | *Alabama,* | Dr. Malone. |
| Abel Carey, | *Ohio,* | Practitioner. |
| Erastus S. Close, | *Ohio,* | Prfs. Harrison & McDowell. |
| Jonathan W. Coffee, | *Ohio,* | Dr. Fisher. |
| Benjamin F. Crittenden, | *Alabama,* | Prfs. Harrison & McDowell. |
| Thomas W. Colescott, | *Indiana,* | Dr. Haymond. |
| James M. Conduitt, | *Illinois,* | Dr. Conduitt. |
| **D** | | |
| Eli Dayton, | *Ohio,* | Prfs. McDowell & Harrison. |
| John Dawson, | *Ohio,* | Dr. Winmans. |
| Edwin H. Davis, | *Ohio,* | Practitioner. |
| Alexander Dunlap, | *Ohio,* | Drs. McCaraugh & Dunlap. |
| Benjamin Dennis, | *Mississippi,* | Resident Graduate. |
| **E** | | |
| John E. Ely, | *Ohio,* | Prf. Gross. |
| David M. Elliott, | *Ohio,* | Dr. Brower. |
| Peter M. Enders, | *Kentucky,* | Prf. Gross. |
| Jacob P. Epperson, | *Alabama,* | Prfs. Harrison & McDowell. |
| John Evans, | *Ohio,* | Dr. Fisher. |
| Thomas Edwards, | *Alabama,* | Dr. Crook. |
| **F** | | |
| Benjamin W. Foster, | *Ohio,* | |
| Roderick C. Fox, | *Ohio,* | Dr. Dodge. |
| George F. Foot, | *Ohio,* | Dr. Howard. |
| Carey H. Fry, | *Kentucky,* | Dr. Fry. |
| Seth W. Fuller, | *Ohio,* | Dr. Cotton. |
| William M. Finley, | *Tennessee,* | Dr. McCorkle. |

| | | |
|---|---|---|
| **G** | | |
| Edward O. Goodwyn, | *Virginia,* | Hrfs. Harrison & McDowell. |
| Daniel Grosvenor | *New England,* | |
| Jonathan P. Grosvenor, | *New England,* | |
| John Groff, M. D. | *Ohio,* | |
| Charles P. Gage, M. D. | *Ohio,* | |
| Elias Garst, | *Ohio,* | Prfs. Harrison & McDowell. |
| John H. Grant, | *Kentucky,* | Prf. Gross. |
| Tarleton W. Graves, | *Virginia,* | Practitioner. |
| **H** | | |
| Robert C. Hamill, | *Ohio,* | Dr. Templeton. |
| Ambrose W. Henley, | *Ohio,* | Dr. McGuire. |
| Samuel W. Hankin, | *Tennessee,* | |
| Edmund Harrell, | *Indiana,* | Prfs. Harrison & McDowell. |
| William J. P. Haughton, | *Alabama,* | Prf. Gross. |
| James T. Houston, | *Ohio,* | Prfs. Harrison & McDowell. |
| Elijah M. Hussey, | *Alabama,* | Prfs. Harrison & McDowell. |
| Anson G. Henry, | *Illinois,* | Practitioner. |
| **J** | | |
| John B. Jewett, | *Ohio,* | Practitioner. |
| Eli B. Jewett, | *Ohio,* | Practitioner. |
| Benjamin W. Johnson, | *Ohio,* | |
| **K** | | |
| Charles L. Kendall, | *Ohio,* | |
| Edward Kimball, | *Ohio,* | Prfs. McDowell & Harrison. |
| Robert Kincaid, | *Ohio,* | |
| John C. Kirkpatrick, | *Tennessee,* | Dr. McCorkle. |
| **L** | | |
| Morgan H. Litzenburg, | *Ohio,* | Dr. Russell. |
| George Light, | *Ohio,* | Prfs. Harrison & McDowell. |
| Charles C. Little, | *Ohio,* | Dr. Hastings. |
| **M** | | |
| Robert Morehead Mines, | *Ohio,* | Prfs. Harrison & McDowell. |
| Francis McCrackin, | *Ohio,* | Prfs. Harrison & McDowell. |
| Robert R. McIlwayne, | *Ohio,* | Prfs. Harrison & McDowell. |
| James W. McCrady, | *Pennsyivania,* | Dr. Wylie, |
| Joseph L. McCay, | *Pennsylvania,* | Dr. Culbertson. |
| Robert Merrill, Jr. | *Ohio,* | |
| C. V. McMillen, | *Ohio,* | Dr. Robertson. |
| William H. H. B. Minor, | *Ohio,* | Dr. Paramore. |
| James R. Mershon, | *Ohio,* | |
| Jacob P. Morris, | *Kentucky,* | Dr. J. Mines. |
| Wm. M. Maxwell, M. D. | *Virginia,* | |
| Robert McJimsey, | *Mississippi,* | Drs. Pasley & Huggins. |
| Franklin J. Malone, | *Alabama,* | Prfs. Harrison & McDowell. |
| Jonathan McDonald, | *Alabama,* | Practitioner. |
| John McNeill, | *Alabama,* | Practitioner. |
| Eli T. Mendenhall, | *Alabama,* | Dr. Smith. |
| **N** | | |
| Israel T. Nicklin, | *Virginia,* | Practitioner. |
| Allen T. Noe, | *Kentucky,* | Dr. Bowling. |

| | | |
|---|---|---|
| **P** | | |
| John W. Pickett, | *Ohio,* | |
| Henry Pearce, | *Ohio,* | Prfs. Harrison & McDowell. |
| William Peyton, | *Kentucky,* | Prfs. Harrison & McDowell. |
| Alexander A. Patteson, | *Kentucky,* | Dr. Marshall. |
| John Parkes, | *Virginia,* | Prof. Rives. |
| Charles A. Pope, | *Alabama,* | Drs. Fearn & Erskine. |
| James A. L. Purdom, | *Alabama,* | Drs. Fearn & Erskine. |
| William J. Peck, | *Indiana,* | Prfs. Harrison & McDowell. |
| Ferdinand S. Petway, | *Tennessee,* | Dr. McKinney. |
| Alexander H. Petit, | *Mississippi,* | Practitioner. |
| **R** | | |
| Townsend Ryan, | *Ohio,* | |
| Sanderson H. Rodgers, | *Ohio,* | Dr. Dodge. |
| Joseph Redhead, | *Ohio,* | Prof. Gross. |
| Richard C. Ratcliff, | *Alabama,* | Dr. Sterritt. |
| Samuel Reed, | *Indiana,* | Dr. Reed. |
| Pleasant B. Robinson, | *Alabama,* | Dr. Malone. |
| **S** | | |
| John B. Smith, | *Ohio* | Dr. Riddell. |
| Robert B. Scott, | *Mississippi,* | Prfs. Harrison & McDowell. |
| Samuel M. Smith, | *Indiana,* | Dr. Morrison, |
| John C. Spotswood, | *Alabama,* | Practitioner. |
| Edward A. Scruggs, | *Alabama,* | Drs. Fearn & Erskine. |
| Julias Sharp, | *Ohio,* | Dr. Sams. |
| William Smith, | *New York,* | |
| **T** | | |
| David L. Talbott, | *Ohio,* | Prfs. Harrison & McDowell. |
| Francis W. Todd, | *Illinois,* | Prfs. Harrison & McDowell. |
| John W. Tucker, | *Ohio,* | Dr. Peck. |
| Norton S. Townshend, | *Ohio,* | Dr. Howard. |
| Benjamin Thornton, | *Kentucky,* | Dr. Lindsay. |
| **V** | | |
| Isaac Van Eaton. | *Ohio.* | Prfs. Harrison & McDowell. |
| **W** | | |
| David Wilson, | *Ohio,* | Dr. Dodge. |
| Benjamin W. Wood, | *Kentucky,* | Dr. Marshall. |
| Madison Williams, | *Kentucky,* | Dr. Roberts. |
| Madison G. Walker, | *Indiana,* | Dr. Richmond. |
| James W. Wilson, | *Indiana,* | Dr. Jennings. |
| Wilkins Watson, | *Illinois,* | Practitioner. |
| Isaac H. Wright, | *Kentucky,* | Dr. S. K. Sharp. |
| Francis A. Williamson, | *N. Carolina,* | Dr. Tresvant. |
| Oliver M. Wozencraft, | *Tennessee,* | Prfs. Harrison & McDowell. |
| **Y** | | |
| Phillip Young, Jr. | *Ohio,* | Dr. Gross. |
| William H. Yarbrough, | *Alabama,* | Prfs. Harrison & McDowell. |
| John A. Young, | *Ohio,* | Dr. Martin. |

### RECAPITULATION.

| | | | |
|---|---|---|---|
| Ohio, - - - - | 57 | Alabama, - - - - | 18 |
| Kentucky, - - - | 14 | Indiana, - - - - | 9 |
| Tenessee, - - - | 5 | Virginia, - - - - | 6 |
| Pennsylvania, - - - | 3 | Illinois, - - - - | 4 |
| Mississippi, - - - | 4 | New England, - - - | 2 |
| Missouri, - - - | 1 | New York - - - | 1 |
| North Carolina, - - | 1 | | |
| | | Total, | 125 |

### GRADUATES.

At the third annual Commencement, holden on March 3d, 1838, the Degree of Doctor of Medicine was conferred on the following gentlemen:

### OHIO.

*Abel Carey*, Thesis on Scarlet Fever.
*Erastus S. Close*, on Blood letting.
*Edwin H. Davis*, on Electricity as a cause of Epidemics.
*Eli Dayton*, on Blood letting.
*John Evans*, on Medullary Sarcoma.
*Elias Garst*, on Acute Gastritis.
*John B. Jewett*, on the Blood.
*Edward Kimball*, on Arachnitis.
*Joseph Redhead*, on Lesions of the Pleura.
*John A. Young*, on the relations of the nervous and vascular systems in Intermittent Fever.

### ALABAMA.

*William J. P. Haughton*, Thesis on the General and Pathological Anatomy of the Arteries.
*John McNeill*, on Marsh Miasmita.
*Jonathan McDonald*, on External Local Irritants.
*John C. Spotswood*, on Leucorrhœa.

### KENTUCKY.

*John S. Alexander*, on the Morbid Anatomy of the Intestinal Mucous Membrance.
*John H. Grant*, on the Pathological Anatomy, and treatment of Tubercular Consumption.
*William Peyton*, on Inflammation.

### ILLINOIS.

*Anson G. Henry*, on the Diseases of Sangamon Co. Illinois.
*F. Walter Todd*, on the Milk Sickness.
*Wilkins Watson*, on Gonorrhœa.

### INDIANA.

*Ziba Casterline*, on Amenorrhœa.
*Thomas W. Colescott*, on a recent Epidemic fever at Brookville, Indiana.

### MISSISSIPPI.

*Alexander H. Petit*, on Miasmata.

The Honorary Degree of Doctor of Medicine was likewise conferred on *Samuel Parsons*, of Columbus, and *Daniel Milliken*, of Hamilton, in the state of Ohio.

JAMES B. ROGERS, *Dean*.

### INFORMATION TO STUDENTS OF MEDICINE.

The session formally opens on the last Monday of October, annually, and closes on the day preceding the public commencement in March, but DISSECTIONS, under the direction of Dr. Trimble and a preliminary course of OSTEOLOGY, by Professor McDowell, commence on the first Monday of October without any additional charge. Six Lectures are delivered daily, to the end of February, when the number is reduced to four, and the examinations commence. An hour every other day is devoted by the Professors of the PRACTICE of PHYSIC and of SURGERY, to the study of cases in the *Marine Hospital*, and the *Cincinnati Eye Infirmary*. All who intend to graduate, are required to take the Dissecting ticket once, and the Hospital ticket once—the latter in the graduating session. It is optional with the student to take or omit the Library ticket. The price of the ticket of each Professor is $15—Matriculation $2—Dissections $10—Hospital $5—Library $3—Graduation $20. Two full courses, the last in this institution, or four years practice and one course, are necessary to an examination for a degree. A candidate must register his name with the Dean by the first of February, and hand in his Thesis by the 20th of that month. The examinations commence on the 1st of March, and the public Commencement is held as soon as they are concluded.

The purchases of Professor Parker in Europe, and of Professor Rogers in Baltimore and Philadelphia, during the past year, have greatly extended the APPARATUS, MUSEUM, and LIBRARY of the institution.

### SUMMER INSTRUCTION.

At the close of the session, the rooms for Practical Anatomy are reopened by the *Demonstrator*, and kept open for a month. Students are likewise admitted to the practice in the Hospital, which is visited every morning at 8 o'clock, by two of the Professors. Lectures will be delivered daily in the College edifice, opposite the Hospital, from the end of the prescribing hour till 11 o'clock. Price of the ticket to both, $10.

The Library embraces 1300 volumes: the ticket to which, for the eight months vacation, is $5.

Comfortable boarding and lodging may be had at from $2,50 to $3,50 per week.

JAMES B. ROGERS,
*Dean of the Faculty.*

CINCINNATI COLLEGE, March, 3d, 1838.

